# Current practices and perceived effectiveness of alternative behavioral management techniques for pediatric dental anxiety: a cross-sectional survey of dentists in Spain

**DOI:** 10.3389/fdmed.2026.1783025

**Published:** 2026-03-25

**Authors:** Daniela del Toro-Alcántara, Marcela Arenas González, Ángel Luis Formoso Veloso, Carolina Caleza Jiménez, María José Barra Soto, María Biedma Perea, David Ribas-Perez

**Affiliations:** Faculty of Dentistry, Universidad de Sevilla, Seville, Spain

**Keywords:** alternative techniques, behavioral management, dental anxiety, fear of the dentist, pediatric dentistry

## Abstract

**Objectives:**

Fear of the dentist and associated anxiety in pediatric patients can negatively impact dental care and oral health. Traditional behavioral management techniques, such as physical restraint or sedation, are increasingly debated and rejected. The main objective of this study was to analyze the level of knowledge, use, and perceived effectiveness of alternative behavioral management techniques among practicing dentists in Spain.

**Materials and methods:**

A cross-sectional study was conducted with 144 dental practitioners from Seville, Spain, predominantly female (77.8%) and aged 25–35 years (44.4%). Participants represented various specialties, with general dentistry being the most common (51.4%). Data were collected regarding the frequency of use, knowledge, and perceived effectiveness of behavioral management techniques for pediatric patients.

**Results:**

Across all specialties, the most frequently reported behavioral management technique was tell-show-do, particularly among general dentists, orthodontists, and pediatric dentists. Endodontists primarily favored positive reinforcement, whereas oral surgeons/implantologists reported higher use of pharmacological management. Use of physical restraint was minimal and limited to pediatric dentistry.

**Conclusions:**

The study highlights the need to enhance training in emotional management and alternative behavioral management techniques to prevent future trauma and reduce the likelihood that children will avoid dental care due to anxiety. Additionally, adapting the clinical environment to be more child-friendly is important to support effective behavioral management.

**Clinical relevance:**

Improving dentists' knowledge and use of alternative behavioral management strategies can reduce pediatric dental anxiety, improve patient cooperation, and promote long-term oral health by encouraging regular dental visits.

## 1 Introduction

Pediatric dentistry is currently recognised as a part of dentistry dedicated to the prevention and maintenance of oral health in pediatric patients, as well as to the provision of treatments aimed at restoring lost oral health from birth through adolescence (1). Successful treatment in pediatric patients requires knowledge of behavioral management strategies (2). Therefore, dental practitioners should be familiar with the available techniques and select the most appropriate approach according to each child's individual characteristics (3, 4).

### 1.1 Current situation

At present, the term “anxiety” is used in a wide range of contexts and across multiple disciplines, including healthcare. Anxiety is defined as an emotional state characterised by feelings of unease, fear or worry in response to perceived threatening situations (5). In the field of dentistry, this emotional state holds significant relevance, as numerous studies have observed and demonstrated that a high percentage of the pediatric population suffers from dental anxiety (ranging from 3% to 43%) (5, 6) that is, a marked fear or distress associated with dental visits. In pediatric dentistry, the distinction between fear and anxiety has important clinical implications. Fear can be understood as an objective, reactive emotional response to an identifiable threat within the dental setting, and its expression varies significantly depending on the age of the patient undergoing treatment (2).

These conditions directly affect clinical practice by causing children to avoid dental visits. This scenario contribute to a decrease in overall dental care increasing the risk of long-term oral health issues (7). These may include the development of harmful habits that result in skeletal malocclusions, which could otherwise be intercepted during childhood to prevent future dental misalignments.

For decades, various techniques have been employed to control and manage patient behavior, including physical restraint methods such as the Hand-Over-Mouth Technique (HOM), and immobilisation of the hands, feet and head. Pharmacological sedation has also been used in cases where anxiety or uncooperative behavior hindered the proper execution of treatment (8). However, the use of such methods has become increasingly controversial, raising concerns about their ethical implications and their potential impact on the patient's emotional well-being (9, 10).

Parents (and increasingly dental professionals) tend to oppose the use of these techniques. This shift is partly due to a growing awareness of children's rights and emotional development, and partly to a protective parenting style, both of which have contributed to a decline in their clinical application (9).

Parents therefore play a significant role in the clinical context of pediatric dentistry. Dental anxiety can be transmitted between generations. Parents' attitudes toward dental treatment strongly influence how children perceive dental visits. Adults, whether consciously or unconsciously, often project their own fears onto the child, thereby predisposing them to anxiety and fear in dental settings (11, 12).

In response to this situation, there has been growing interest in the use of alternative behavioral management techniques in pediatric dentistry. These aim to reduce dental anxiety without resorting to methods that may be ethically questionable or uncomfortable for the patient, parents or the practitioner. Among the strategies that have shown promising results are music therapy, virtual reality glasses, clinical hypnosis, aromatherapy, and other therapeutic approaches such as breathing relaxation techniques. All of these seek to enhance the child's experience in the dental environment (13). Evidence suggests that these techniques improve relaxation and facilitate dental treatment.

### 1.2 Dental anxiety

As defined in the previous section, anxiety is an emotional state that may or may not be linked to a specific stimulus. In this context, it is indeed associated with a particular situation-namely, the dentist, dental procedures, or any circumstance related to the dental clinic.

Dental phobia (14) (a severe fear or panic reaction to dental settings) is a daily challenge for dental professionals. Therefore, it is essential to address and manage dental anxiety from an early age to reduce the risk of developing full-blown phobias in adulthood, which are typically more difficult to treat due to their subconscious nature and may require intervention from mental health professionals.

Different levels of anxiety can be identified depending on their intensity (15). At the no anxiety level, the patient is completely relaxed and shows no signs of distress. When experiencing mild anxiety, the patient may begin to feel slight unease or nervousness, particularly in response to certain therapeutic or diagnostic procedures. Moderate anxiety is characterized by increased nervousness, often stemming from uncertainty. At this stage, the patient may become apprehensive, which can interfere with their ability to cooperate during treatment. In cases of high anxiety, the patient exhibits a severe emotional response, including intense fear or panic, which can make it difficult or even impossible to carry out dental procedures. In such situations, specific management techniques must be used to address and reduce the patient's anxiety effectively.

Children's ability to cope with fear and their emotional responses vary greatly. Thus, it is vital to tailor behavioral management strategies to the individual needs of each child (11). However, all techniques share a common goal: to reduce stress-inducing stimuli an essential aspect of achieving successful outcomes. Stimuli are processed through the senses, which transmit signals to the brain that elicit a response.

Focusing on the senses involved in the dental experience, several types of stressors can be identified. Visual stimuli include the sight of objects such as needles, dental burs, sharp instruments, and the handpiece, all of which can provoke anxiety. Auditory stimuli also play a significant role; once seated in the dental chair, children are exposed to characteristic sounds like the turbine, air compressor, and other loud equipment, which can have a negative emotional impact. Tactile sensations are another source of stress, as intraoral procedures often involve instruments like the handpiece or micromotor that generate vibrations, potentially causing discomfort and unease. Finally, olfactory stimuli can trigger stress responses, particularly when certain smells are linked to negative past experiences. Common odors in the clinic, such as those from eugenol or dental adhesives, may become subconsciously associated with discomfort or fear (15).

It is crucial to raise parents' awareness of the important role they play in mitigating dental anxiety (11, 15). Dentists should guide and educate caregivers to avoid negative comments about past experiences, to foster a positive attitude towards dental care, and to encourage their children to view visits as enjoyable experiences. Communication should be calm and optimistic, especially when discussing upcoming procedures.

Therefore, the management of child behavior in dental settings should not be limited to the patient alone; it must also involve the education and engagement of parents and guardians (16, 17).

### 1.3 Conventional techniques

Dentists, particularly those specialising in pediatric dentistry, have developed various techniques over the years aimed at preventing dental anxiety. These methods focus on reducing anxiety and discomfort secondarily, thereby helping the child remain relaxed and comfortable during the consultation.

Between these techniques these, are the most used: Tell-Show-Do, Positive Reinforcement, Desensitisation, Modelling, Voice Control, Physical Restraint and Premedication or General Anestesia (7, 18).

### 1.4 Alternative techniques for managing dental anxiety

#### 1.4.1 Aromatherapy

Aromatherapy utilises essential oils extracted from flowers, roots, or trees, which contain aromas that positively influence behavior. These oils are dispersed into the clinic environment via diffusers, where the patient and (even those in the waiting room) can inhale the scent, producing psychological and physiological calming effects. This natural, non-invasive method is increasingly applied in dentistry to promote relaxation and reduce anxiety (19–22).

Essential oils can be administered in various ways to support therapeutic outcomes. Topical application includes methods such as massages or oral ingestion, either through infusions or drops placed under the tongue. Olfactory administration involves inhalation or atmospheric diffusion, allowing the aromatic compounds to influence the body through the sense of smell.

Different essential oils are selected based on their specific properties, such as relaxation, analgesia, anti-inflammatory, or antibacterial effects. In the context of dental anxiety, commonly used essential oils include lavender, chamomile, geranium, rose, sage, oregano, and neroli. These oils are believed to help alleviate anxiety and promote a sense of calm.

Research, such as the study by Gedney et al. (21), indicates that while the inhalation of aromatic oils like lavender and rosemary may not produce measurable analgesic effects, patients often report subjective benefits. These include reduced pain intensity and unpleasantness, with lavender showing particularly positive responses in terms of perceived comfort.

#### 1.4.2 Music therapy

Music therapy is widely used in pediatric dentistry as a tool to help children and adolescents relax, disconnect from the clinical environment, and reduce anxiety. Dentists often tailor the music to suit each patient's preferences, choosing from options such as ambient music, children's songs, calming melodies, or even narrated stories.

Research suggests that music may reduce anxiety during dental treatment, although its effect on pain is limited. Music encourages children to close their eyes and concentrate on the sounds, helping to distract them from the ongoing procedure. It also helps to mask unpleasant clinical noises, such as those produced by the dental handpiece, which are commonly associated with fear or discomfort. Additionally, music naturally induces a state of relaxation in many patients, although the degree of its effectiveness can vary depending on individual musical preferences (22).

#### 1.4.3 Hypnosis

Hypnosis is a wakeful state of focused attention in which an individual becomes deeply absorbed in internal experiences such as thoughts, emotions, and sensations. It can occur naturally, be induced by another person, or be self-induced. In dental practice, hypnosis is employed as a supportive technique to manage dental anxiety, treat specific phobias, control pain during procedures, and enhance patient tolerance to orthodontic appliances. It also serves as a useful adjunct to nitrous oxide sedation and can aid in modifying oral habits, as well as reducing nausea or assisting with smoking cessation.

There are several advantages to using hypnosis in dentistry. It does not require any specialized equipment although proper training for the practitioner is essential. The patient remains conscious throughout the process, and there are no pharmacological side effects. Additionally, hypnosis can be effectively combined with inhalation sedation for enhanced outcomes.

However, there are certain limitations to its use. Successful application of hypnosis depends on the patient's ability to understand instructions, possess age-appropriate cognitive development, and demonstrate emotional stability. A supportive social environment is also important for achieving optimal results (23–25).

#### 1.4.4 Relaxation and breathing techniques

Relaxation techniques can help patients feel calm and reduce anxiety. The benefits of this approach include lowering anxiety levels, improving the ability to cope with stress, stabilizing heart and respiratory functions, enhancing concentration and reflexes, increasing positive thinking and self-confidence, and reducing muscle tension (26).

Rhythmic breathing works by counteracting the “fight or flight” response, helping to focus the mind and calm the nervous system. Clinical studies have demonstrated that slow, deep breaths-held for about 5 s and then exhaled slowly over a period of 2–4 min can reduce heart rate and induce a state of relaxation. This breathing technique is easy to teach in clinical settings and can be practiced at home before stressful appointments to help patients better manage their anxiety (27).

#### 1.4.5 Audiovisual methods and virtual reality (VR)

Technological advances have led to the use of VR in pediatric dentistry to manage anxiety. VR offers multiple benefits as it allows uninterrupted treatment. It is comfortable and engaging for patients. It also distracts from painful stimuli and if equipped with sound, reduces dental chair noises (28–30).

Studies comparing music therapy with audiovisual distraction (e.g., Prabhakar et al.) found VR more effective in reducing anxiety in children aged 4–8. VR headsets with high-definition LCD screens block external stimuli and immerse the patient fully, preventing them from seeing dental instruments, thus lowering fear. These devices pose no radiation risk (29).

However, VR is not suitable for all patients, especially those who feel claustrophobic or prefer to maintain control during treatment. It is less recommended for overly vigilant children.

This study aimed to analyze the clinical use of alternative behavioral management techniques in pediatric dentistry. We also wanted to analyse the perceived effectiveness of these techniques. Perceived effectiveness has been defined as the clinician's subjective assessment of the extent to which behavioral management techniques resulted in observable improvement in patient behavior and facilitated the delivery of dental treatment. This construct reflects the practitioner's professional judgment regarding reductions in patient anxiety, increased cooperation, decreased disruptive behaviors, and overall enhancement of clinical manageability, independent of objective behavioral metrics.

## 2 Materials and methods

### 2.1 Study design

This study is observational, cross-sectional, and descriptive in nature. A survey was conducted to collect sufficient information to analyse and determine the primary and specific objectives of the project, aiming to obtain robust conclusions. The conventional techniques most frequently employed in behavior management were selected, together with alternative approaches that, according to the literature, have shown higher levels of acceptance among dental practitioners (13).

### 2.2 Sample and population

The study population consisted of general dental practitioners currently practising. This group included dentists specialising in Pediatric Dentistry as well as those specialising in other fields such as periodontics or prosthodontics who nonetheless treat pediatric patients.

Participants were selected through non-probabilistic convenience sampling from a list of dentists offered publicly by the regional government of Andalucia. Participation was voluntary, anonymous, and without any financial compensation. There were no exclusion criteria.

According to the General Dental Council of Andalucía, in Seville, there are 4,075 registered dentists. To ensure representativeness, the sample size was calculated considering the population size, confidence level, and margin of error, resulting in a required sample of 145 dentists.

### 2.3 Evaluation instruments

A digital questionnaire developed by Dr Begoña Bartolomé Villar was used (13). The questionnaire assessed dentists' knowledge, use, perceived effectiveness, and interest in alternative behavioral management techniques.

The instrument demonstrated established content validity and construct validity, confirming that the items adequately represented the theoretical dimensions of behavioral management outcomes. The questionnaire included categorical response options that allowed practitioners to report perceived behavioral improvement, no improvement, or non-application of the techniques.

The survey was administered via Google Forms to facilitate dissemination and accessibility across multiple devices. It comprised 18 questions, including sociodemographic variables (sex, age, and speciality), closed-ended questions, multiple-choice items and Likert scales (Supplementary Material).

### 2.4 Statistical analysis

Categorical variables were summarized using absolute frequencies and relative percentages. To explore factors associated with the adoption of alternative behavioral management strategies, a series of logistic regression models were fitted. The dependent variables corresponded to the implementation of specific alternative techniques in clinical practice (play area availability, pediatric-themed operatories, use of children's pattern uniforms, audiovisual tools, music therapy, aromatherapy, and breathing relaxation techniques), each coded as a binary outcome variable (implemented vs. not implemented).

Independent variables included dental specialty, age group, and gender. Categorical predictors were entered into the models using dummy coding, with general dentistry serving as the reference category for specialty, age ≤35 years as the reference category for age group, and male practitioners as the reference category for gender.

Because some specialties had relatively small sample sizes and several outcome categories exhibited low-frequency responses, Firth's bias-reduced logistic regression was applied to reduce small-sample bias and prevent separation problems that may arise in conventional maximum likelihood logistic regression. Model results are reported as adjusted odds ratios (ORs) with corresponding 95% confidence intervals (CIs). All statistical analyses were conducted using R software version 4.5.1 (R Foundation for Statistical Computing).

## 3 Results

### 3.1 Participant characteristics

The study cohort comprised 144 dental practitioners from Seville, Spain, with demographic and professional characteristics detailed in Table 1. The sample was predominantly female (*n* = 112, 77.8%), with a modal age distribution of 25–35 years (*n* = 64, 44.4%). Notably, 30.6% (*n* = 44) were under 25 years. General dentistry constituted the largest specialty group (*n* = 74, 51.4%), followed by pediatric dentistry (*n* = 31, 21.5%) and orthodontics (*n* = 16, 11.1%). Some specialty subgroups in the sample were relatively small, particularly prosthodontists (*n* = 3) and endodontists (*n* = 9). Consequently, comparisons involving these groups should be interpreted with caution, as the limited sample sizes may reduce statistical power and increase the variability of the estimated proportions.

**Table 1:** Demographic and professional characteristics of study participants.

Characteristic	*N* = 144^a^
Age, years	
<25	44 (30.6%)
25–35	64 (44.4%)
36–45	20 (13.9%)
46–55	13 (9.0%)
>55	3 (2.1%)
Gender	
Male	32 (22.2%)
Female	112 (77.8%)
Main occupation	
General dentistry	74 (51.4%)
Endodontics	9 (6.3%)
Orthodontics	16 (11.1%)
Pediatric dentistry	31 (21.5%)
Prosthodontics	3 (2.1%)
Oral surgery/implantology	11 (7.6%)

^a^ *n* (%).

### 3.2 Conventional behavior management techniques

**Figure 1:**
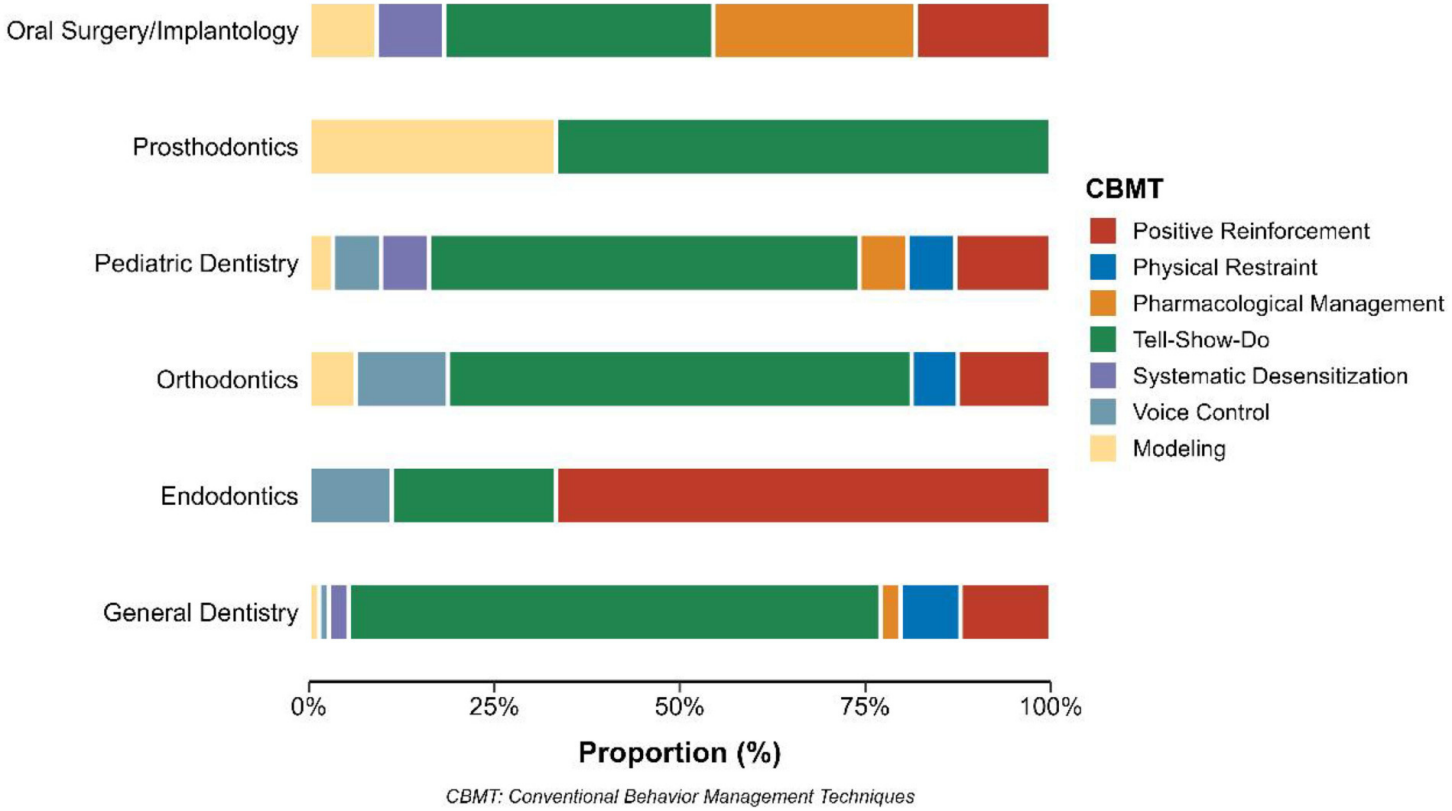
Distribution of conventional behavioral management techniques used by dentists according to specialty. The figure illustrates the primary behavioral management technique reported by practitioners in each dental specialty, including Tell-Show-Do, positive reinforcement, voice control, modeling, pharmacological management, physical restraint, and systematic desensitization.

The distribution of primary conventional behavioral management techniques varied across dental specialties. Among general dentists (*n* = 74), tell-show-do was the most frequently reported technique (71.6%), followed by positive reinforcement (12.2%). Endodontists (*n* = 9) predominantly used positive reinforcement (66.7%) and voice control (11.1%). Orthodontists (*n* = 16) primarily employed tell-show-do (62.5%), with voice control (12.5%) and modeling (6.2%) as secondary techniques. Pediatric dentists (*n* = 31) most frequently reported tell-show-do (58.1%), while physical restraint, pharmacological management, and systematic desensitization each accounted for 6.5% of responses. Prosthodontists (*n* = 3) used tell-show-do (66.7%) and modeling (33.3%). Oral surgeons/implantologists (*n* = 11) reported tell-show-do (36.4%) as the primary technique, followed by pharmacological management (27.3%) and positive reinforcement (18.2%). Physical restraint was not reported by endodontists, prosthodontists, or oral surgeons/implantologists (Figure 1) (Supplementary Table S1). However, the interpretation of specialty-specific estimates should be made cautiously due to the limited number of participants in certain specialty groups.

### 3.3 Alternative behavioral management techniques

The use of alternative therapies varied across dental specialties (Table 2). Pediatric dentistry demonstrated the highest implementation rates for environmental modifications, with 77.4% (*n* = 24) of practitioners maintaining play areas and 80.6% (*n* = 25) featuring pediatric-themed operatories. Music therapy was most prevalent among pediatric dentists (93.5%, *n* = 29) and orthodontists (87.5%, *n* = 14). Breathing relaxation techniques were widely adopted by pediatric dentists (93.5%, *n* = 29) and oral surgeons/implantologists (81.8%, *n* = 9). Audiovisual tools were employed by 67.7% (*n* = 21) of pediatric dentists compared to 27.3% (*n* = 3) of oral surgeons. Aromatherapy usage was highest among orthodontists (18.8%, *n* = 3) and pediatric dentists (22.6%, *n* = 7), while prosthodontists reported no aromatherapy use.

**Table 2:** Alternative therapy implementation by dental specialty.

Alternative therapy	General dentistry	Endodontics	Orthodontics	Pediatric dentistry	Prosthodontics	Oral surgery/implantology
	*N* = 74^a^	*N* = 9^a^	*N* = 16^a^	*N* = 31^a^	*N* = 3^a^	*N* = 11^a^
Play area	27 (36.5%)	2 (22.2%)	9 (56.3%)	24 (77.4%)	1 (33.3%)	0 (0.0%)
Pediatric-themed operatories	16 (21.6%)	2 (22.2%)	7 (43.8%)	25 (80.6%)	0 (0.0%)	2 (18.2%)
Pyjama color						
Solid color	54 (73.0%)	8 (88.9%)	10 (62.5%)	15 (48.4%)	1 (33.3%)	11 (100.0%)
Traditional white	17 (23.0%)	0 (0.0%)	2 (12.5%)	2 (6.5%)	2 (66.7%)	0 (0.0%)
Children's patterns	3 (4.1%)	1 (11.1%)	4 (25.0%)	14 (45.2%)	0 (0.0%)	0 (0.0%)
Audiovisual tool	34 (45.9%)	5 (55.6%)	8 (50.0%)	21 (67.7%)	1 (33.3%)	3 (27.3%)
Music	45 (60.8%)	5 (55.6%)	14 (87.5%)	29 (93.5%)	1 (33.3%)	8 (72.7%)
Aromatherapy	7 (9.5%)	0 (0.0%)	3 (18.8%)	7 (22.6%)	0 (0.0%)	0 (0.0%)
Breathing relaxation techniques	43 (58.1%)	3 (33.3%)	10 (62.5%)	29 (93.5%)	1 (33.3%)	9 (81.8%)

^a^ *n* (%).

**Figure 2:**
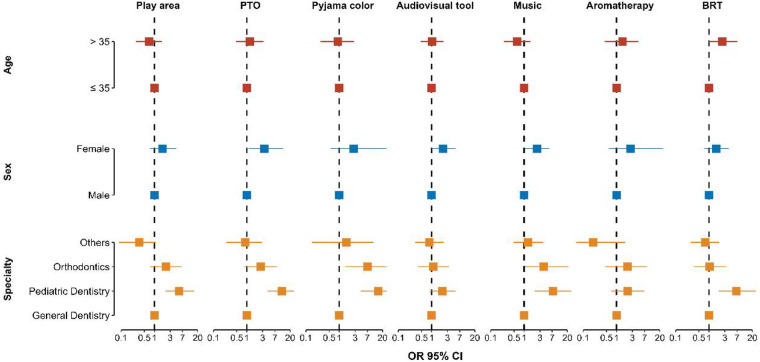
Predictors of the implementation of alternative behavioral management techniques. PTO, pediatric-themed operatories; BRT, breathing relaxation techniques; OR, odds ratio; CI, confidence intervals. Odds ratios (OR) and 95% confidence intervals from Firth's bias-reduced logistic regression models showing associations between practitioner characteristics (dental specialty, gender, and age group) and the implementation of specific alternative behavioral strategies, including pediatric-themed operatories (PTO), breathing relaxation techniques (BRT), children's patterned uniforms, music therapy, and play areas.

Firth's bias-reduced logistic regression models identified specialty-specific patterns as primary predictors of alternative therapy adoption. Pediatric dentists demonstrated significantly greater utilization than general dentists across multiple domains: play areas (OR = 5.50, 95% CI: 2.14–15.62, *p* < 0.001), pediatric-themed operatories (OR = 11.38, 95% CI: 4.23–34.51, *p* < 0.001), children's pattern pyjamas (OR = 14.75, 95% CI: 4.38–63.58, *p* < 0.001), music therapy (OR = 7.42, 95% CI: 2.10–39.79, *p* < 0.001), and breathing relaxation techniques (OR = 6.70, 95% CI: 1.95–35.10, *p* = 0.002). Female practitioners showed increased adoption of pediatric-themed operatories (OR = 3.36, 95% CI: 1.14–12.22, *p* = 0.027) and music therapy (OR = 2.43, 95% CI: 1.04–5.69, *p* = 0.040) compared to males. Orthodontists reported higher use of children's pattern pyjamas (OR = 7.13, 95% CI: 1.54–35.93, *p* = 0.013) and music therapy (OR = 3.89, 95% CI: 1.06–21.21, *p* = 0.040) relative to general dentists. Practitioners aged >35 years utilized breathing relaxation techniques more frequently than younger colleagues (OR = 2.54, 95% CI: 1.01–7.11, *p* = 0.049). No significant predictors emerged for audiovisual tools or aromatherapy adoption (Figure 2) (Supplementary Table S2).

### 3.4 Perceived effectiveness of alternative techniques

Pediatric dentists reported the highest levels of behavioral improvement with 96.8% (*n* = 30) reporting positive behavioral outcomes. Importantly, none of the practitioners within this specialty reported non-application of these techniques, highlighting both systematic implementation and a consistently high level of perceived effectiveness.

Among general dentists, 68.9% (*n* = 51) reported behavioral improvement, indicating a generally favorable perception of effectiveness, although 16.2% (*n* = 12) observed no improvement. In contrast, endodontists demonstrated the greatest variability in perceived effectiveness, with an equal proportion reporting improvement (44.4%, *n* = 4) and no improvement (44.4%, *n* = 4), representing the highest rate of negative outcomes among all specialties.

Orthodontists showed predominantly positive perceived effectiveness, with 68.8% (*n* = 11) reporting behavioral improvement and only 12.5% (*n* = 2) indicating no improvement. Prosthodontists exhibited the highest rate of non-application (66.7%, *n* = 2), suggesting limited integration and potentially lower perceived clinical relevance of behavioral management techniques within this specialty.

**Figure 3:**
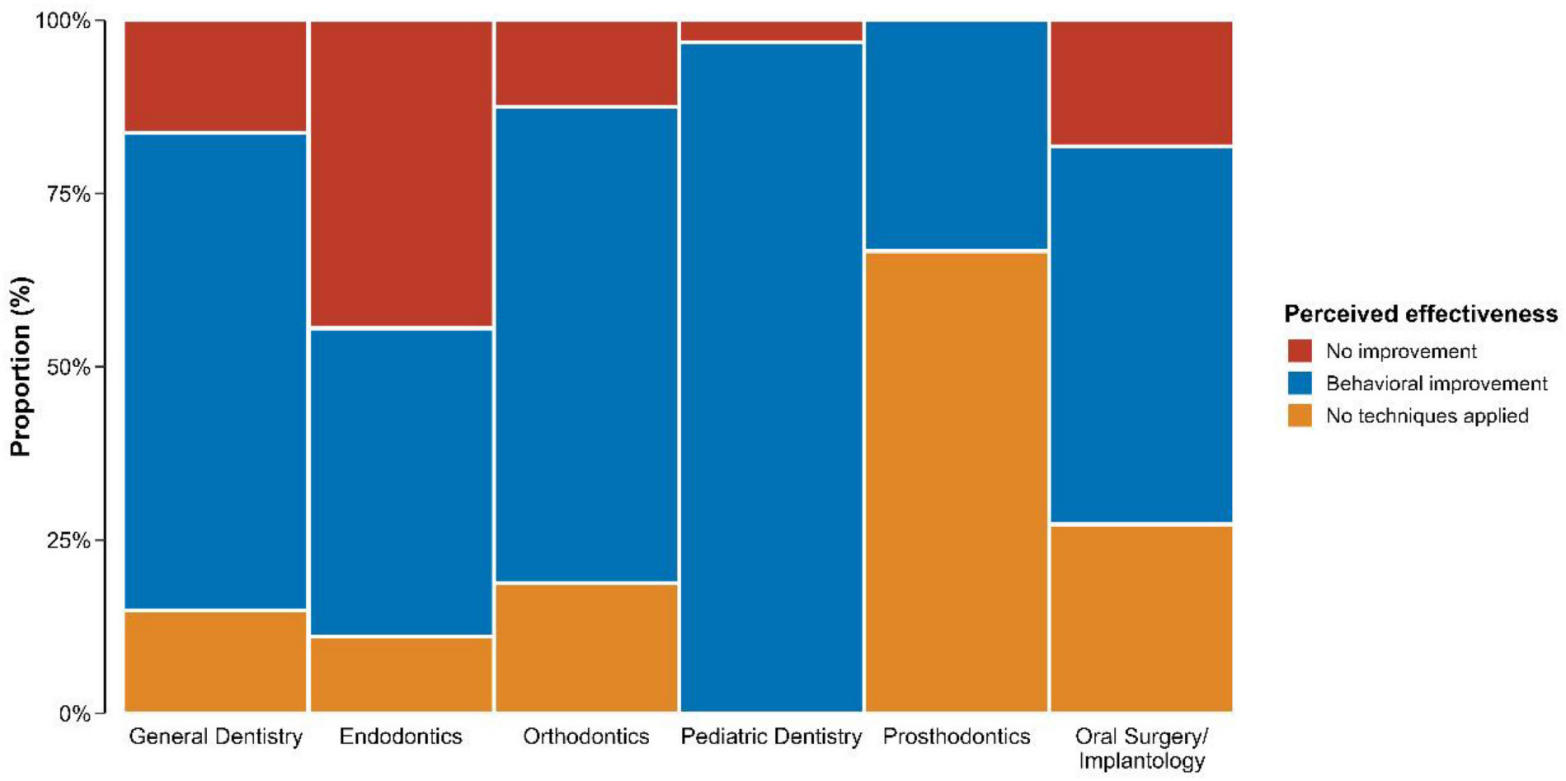
Dentists’ perceived effectiveness of alternative behavioral management techniques by specialty. The figure shows the proportion of practitioners in each dental specialty reporting behavioral improvement, no improvement, or non-application of alternative behavioral management strategies in pediatric patients.

Oral surgeons/implantologists reported behavioral improvement in 54.5% (*n* = 6) of cases, whereas 18.2% (*n* = 2) perceived no improvement, reflecting moderate but comparatively less consistent perceived effectiveness relative to pediatric and general dentists (Figure 3).

**Figure 4:**
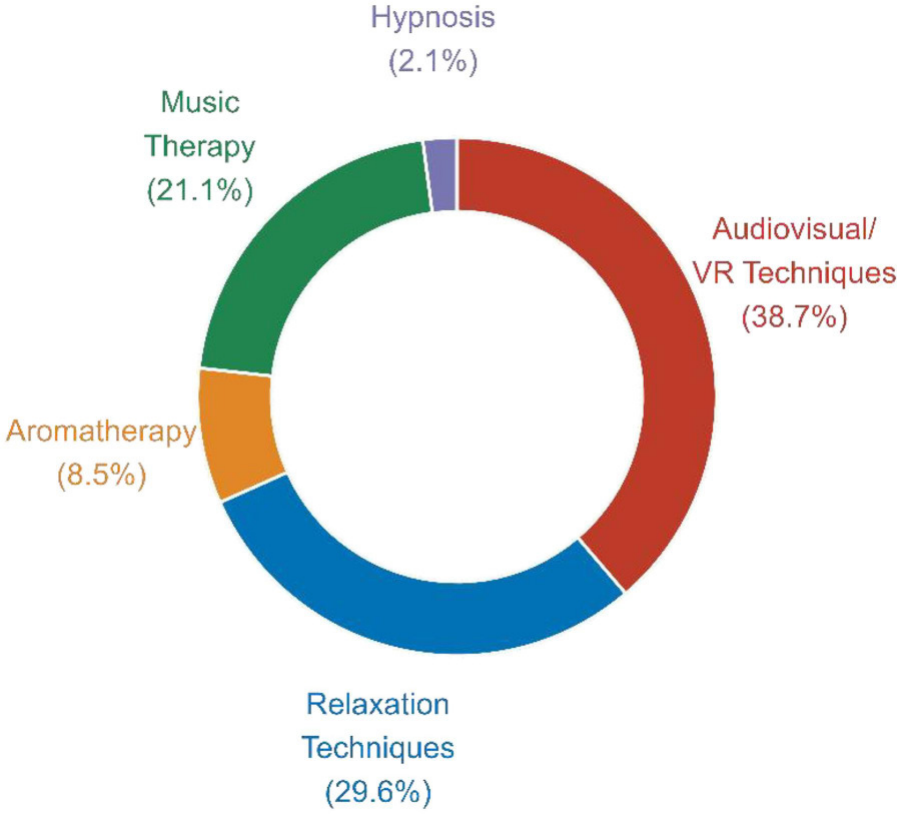
Dentists’ ratings of the most effective alternative behavioral management technique. The figure shows the proportion of respondents who identified audiovisual/virtual reality distraction, relaxation techniques, music therapy, aromatherapy, or hypnosis as the most effective strategy for reducing pediatric dental anxiety.

Among 144 dental practitioners evaluating relative therapeutic effectivness, audiovisual/VR techniques were rated as most effective by 55 respondents (38.7%), representing the highest endorsement across modalities. Relaxation techniques were selected by 42 practitioners (29.6%), followed by music therapy (30 respondents, 21.1%). Aromatherapy received limited recognition (12 respondents, 8.5%), while hypnosis was least frequently endorsed (3 respondents, 2.1%). Two participants (1.4%) did not designate any therapy as superior (Figure 4).

## 4 Discussion

A child's behavior can pose a significant challenge for dental professionals aiming to provide effective treatment. As discussed throughout this study, pediatric patients exhibit varied responses to dental experiences, influenced by multiple factors including their health status, cultural background, parental upbringing styles, age, cognitive level, fear and anxiety, stranger anxiety, pathology, social expectations, and temperament.

As a result, some dentists avoid treating children with difficult behavior. For instance, Ortega et al. (2) and Bartolomé Villar et al. (13) highlighted that the use of alternative techniques remains incipient in many practices, especially where pediatric specialists are absent. Our findings corroborate this, showing that despite awareness of these techniques, their application in daily practice remains limited.

Regarding conventional techniques, the most commonly used method among dentists today is the Tell-Show-Do technique (74.47%). This aligns with Teixeira Antunes et al. (8), who emphasised this method's effectiveness and widespread use over decades due to its simplicity, applicability, and positive outcomes in children with mild to moderate anxiety. Similarly, the systematic review by Gizani et al. (4) concluded that basic techniques such as Tell-Show-Do, positive reinforcement, and voice control remain the most adopted.

Conversely, the least utilised technique, and often chosen as a last resort, is premedication via sedation or general anaesthesia (45.75%). This concurs with observations by Chico et al. (9), who warned of increasing ethical and legal concerns over these interventions, particularly when clear clinical indications or informed parental consent are absent. The decline in usage, also evident in our study, reflects a shift towards more empathetic dentistry prioritising patient wellbeing (31–33).

Among alternative techniques, audiovisual methods including virtual reality were perceived as the most effective by dentists (31.91%). These results reinforce those of Prabhakar et al. (29) and Ram et al. (30), who demonstrated the efficacy of this approach as a distraction tool positively impacting patient cooperation. However, as Adams and Rojas note, economic and logistical barriers may limit their implementation, which is reflected in the low frequency of use observed in the clinics studied. So it was observed a notable “implementation gap”: while audiovisual distractions (VR) were perceived as highly effective, their actual clinical use remains marginal. This discrepancy suggests that logistical and economic barriers limit the use of these techniques.

The second most effective technique reported was relaxation and breathing methods (26.60%), confirming findings by Velasco Zárate (26) and Armfield and Heaton (27), who highlighted its accessibility, low cost, and ease of application in clinical settings.

Music therapy was ranked third (32.98%) in perceived effectiveness. Clinical evidence from authors such as Shih et al. (22) and Aitken et al. (23) supports the benefits of music therapy in reducing anxiety and improving patient attitude, consistent with the perceptions reported here.

In contrast, aromatherapy (38.30%) and hypnosis (43.62%) were considered the least effective techniques. This aligns with Quiroz-Torres and Melgar (20), who reported limited implementation of these methods in dental clinics. The gap between theory and practice may be explained by lack of specific training, perceived difficulty, or unfamiliarity with these techniques.

Regarding child-friendly clinical environments, results reveal a considerable deficiency. Bartolomé Villar et al. (13) emphasise the positive influence of physical environment on reducing dental fear and anxiety, making our findings particularly concerning as such settings are crucial to alleviating fear even before treatment begins.

Statistically significant correlations between speciality and elements such as music type, audiovisual aids, and office decoration suggest a direct link between specialised training and sensitivity to the child's environment in practice. This observation concurs with Bartolomé Villar et al. (12), who noted that pediatric-trained dentists are more inclined to incorporate emotional and environmental approaches in clinical care.

Additionally, the role of parents and guardians is critical in the behavioral management process. Although this study focused on professional perceptions, active family collaboration is essential for successful management. As described by Bartolomé Villar et al. (13), involving parents in the therapeutic process is necessary. Parental attitudes, personal experiences, and approach to dental visits significantly impact the child's perception, as also detailed in intergenerational transmission of dental fear studies (27). Behavior management decisions must be made collaboratively with parents. Professionals may face challenges reaching agreements with parents influenced by media, internet research, or personal adverse dental experiences, but informed consent and consensus are crucial.

This study highlights the need for a comprehensive approach when planning pediatric dental treatment, considering not only technical methods but also emotional, environmental, and communicative aspects. This aligns with Klingberg and Berggren (31), who asserted that not all behavioral problems stem from dental anxiety alone, and that multiple factors influence behavior. Recognising this is vital for designing personalised management strategies in clinical practice (32, 33).

The results show notable differences in perceived behavioral improvement across dental specialties. The near-total perceived effectiveness reported by pediatric dentists (96.8%) compared to the significant “no improvement” rate among endodontists (44.4%) suggests that behavioral outcomes are not solely dependent on the technique used, but are heavily mediated by the nature of the clinical procedure and the provider's specialized training. Endodontic procedures, often associated with acute pain, longer chair times, and higher levels of patient anxiety, likely represent a threshold where non-pharmacological behavioral management techniques reach their limit of effectiveness. This “null finding” in nearly half of endodontic cases underscores the necessity of integrating more advanced pharmacological adjuncts or referral pathways when the complexity of the treatment exceeds the child's coping capacity.

However, certain methodological weaknesses must be acknowledged. The small sample sizes in specific sub-specialties—particularly among prosthodontists (*n* = 3) and endodontists (*n* = 9) limit the statistical power of these comparisons and may lead to an overestimation of the reported rates. These findings should therefore be viewed as preliminary trends rather than definitive epidemiological data. Furthermore, the high “non-application” rate among prosthodontists (66.7%) likely reflects a selection bias in clinical practice, where these specialists may systematically refer pediatric cases to specialists, rather than a failure of the techniques themselves. Future research should utilize standardized, objective behavior scales (e.g., the Frankl Scale) to minimize the subjective bias inherent in practitioners' self-reported “perceptions” of improvement % (34, 35).

It is also interesting to mention in this regard that in Spain there are currently no recognized specialties within dentistry, meaning that any general dentist (or dentist trained in another area of dentistry) can treat children. However, there are specific public health programs for children who are treated by general dentists in the public health system (36).

One limitation of this study could be that it relies on the subjective perceptions of dental professionals rather than objective clinical outcomes (e.g., Frankl Behavior Rating Scale scores or physiological markers of stress) (34, 35). This introduces a potential “social desirability bias,” where practitioners might over-report their use of empathetic techniques (Tell-Show-Do) and under-report more controversial methods like physical restraint or pharmacological interventions to align with current trends.

Another limitation of this study relates to the unequal distribution of participants across dental specialties. Some groups, particularly prosthodontists (*n* = 3) and endodontists (*n* = 9), were represented by relatively small numbers of respondents. As a result, comparisons involving these subgroups should be interpreted cautiously, since limited sample sizes may reduce statistical power and increase the likelihood of unstable estimates. Therefore, the findings related to these specialties should be considered exploratory and warrant confirmation in future studies with larger and more balanced samples.

Finally, the reported “deficiency” in child-friendly environments, despite its known benefits, may reflect a systemic issue in general practices that cater to both adults and children, where spatial and financial constraints limit the prioritisation of pediatric-specific ergonomics. Future research should transition from survey-based perceptions to longitudinal observational studies to validate if these perceived effective techniques translate into measurable improvements in child cooperation and long-term dental anxiety reduction.

## 5 Conclusion

The findings indicate that although dental professionals are broadly familiar with alternative behavior management strategies in pediatric dentistry, their routine implementation remains limited. Reported obstacles include perceived procedural complexity, insufficient training, and financial constraints—particularly regarding audiovisual resources. Tell-Show-Do continues to be the predominant technique, whereas approaches such as sedation are infrequently applied. Among the available alternatives, audiovisual distraction and relaxation techniques appear to have the highest level of acceptance.

Additionally, the study highlights a shortage of child-oriented clinical environments. Elements such as themed treatment rooms or dedicated pediatric waiting areas are relatively rare and tend to be more prevalent in practices where professionals have received specialised training.

## Data Availability

The raw data supporting the conclusions of this article will be made available by the authors, without undue reservation.
