# Supplementary material for: Current practices and perceived effectiveness of alternative behavioral management techniques for pediatric dental anxiety: a cross-sectional survey of dentists in Spain
Source: Front Dent Med. 2026 Mar 25;7:1783025. doi: 10.3389/fdmed.2026.1783025 (PMC13057484; doi:10.3389/fdmed.2026.1783025)
Supplement: Supplementary file 1 [file Table1.docx]

# Alternative Behaviour Management Techniques in Paediatric Dentistry

Survey for undergraduate dissertation. Degree in Dentistry, University of Seville.

Please select the most appropriate answer:

1. Gender

- Female
- Male
- Other

1. Age

- < 25 years
- 25–35 years
- 36–45 years
- 46–55 years
- > 55 years

1. In your professional activity, you mainly practice:

- General Dentistry
- Endodontics
- Orthodontics
- Paediatric Dentistry
- Prosthodontics
- Oral Surgery/Implantology

1. Among the following conventional behaviour management techniques, rank them from most to least frequently used in your practice. Score from 1 (most frequent) to 7 (least frequent):

- Tell–Show–Do
- Positive reinforcement
- Desensitisation
- Modelling
- Voice control
- Physical restraint
- Premedication (sedation/general anaesthesia)

1. Does the clinic where you work have a children’s play area or paediatric waiting room?

- Yes
- No

1. Does the clinic where you work have any surgery room decorated for children?

- Yes
- No

1. Indicate which of the following attire you most frequently wear when treating paediatric patients:

- Traditional white or blue coat/scrubs
- Plain-coloured scrubs
- Scrubs with children’s motifs

1. Do you use any audiovisual media for behaviour management?

- Yes
- No

1. If yes, please specify:

- Television
- Tablets
- Mobile phone
- Audiovisual/virtual reality glasses
- Other:

1. Do you play any type of music in the clinic to reduce dental anxiety?

- Yes
- No

1. If yes, please specify:

- Children’s
- Classical
- Modern
- Other:

1. Do you use any type of aromatherapy to reduce the child’s anxiety?

- Yes
- No

1. If yes, please specify:

- Chamomile
- Lavender
- Geranium
- Other:

1. Do you use or have you ever used hypnosis to reduce anxiety in children?

- Yes
- No

1. Do you use or have you ever used relaxation/breathing techniques to reduce dental anxiety?

- Yes
- No

1. Have you observed that the use of any of these techniques has improved paediatric patient behaviour in the dental clinic?

- Yes
- No
- Have not used any of the above techniques

1. Which of the alternative techniques mentioned do you consider most effective for behaviour management? Rank from 1 (most effective) to 5 (least effective):

- Music therapy
- Aromatherapy
- Audiovisual/virtual reality methods
- Hypnosis
- Relaxation techniques
