# Supplementary material for: Current practices and perceived effectiveness of alternative behavioral management techniques for pediatric dental anxiety: a cross-sectional survey of dentists in Spain
Source: Front Dent Med. 2026 Mar 25;7:1783025. doi: 10.3389/fdmed.2026.1783025 (PMC13057484; doi:10.3389/fdmed.2026.1783025)
Supplement: Supplementary file 2 [file Table2.docx]

# SUPPLEMENTARY MATERIAL

**Supplementary Table S1.** Conventional behavior management techniques by specialty

| **CBMT** | **General Dentistry (n=74)** | **Endodontics (n=9)** | **Orthodontics (n=16)** | **Pediatric Dentistry (n=31)** | **Prosthodontics (n=3)** | **Oral Surgery/Implantology (n=11)** |
| --- | --- | --- | --- | --- | --- | --- |
| Positive Reinforcement | 9 (12.2%) | 6 (66.7%) | 2 (12.5%) | 4 (12.9%) | 0 (0.0%) | 2 (18.2%) |
| Physical Restraint | 6 (8.1%) | 0 (0.0%) | 1 (6.2%) | 2 (6.5%) | 0 (0.0%) | 0 (0.0%) |
| Pharmacological Management | 2 (2.7%) | 0 (0.0%) | 0 (0.0%) | 2 (6.5%) | 0 (0.0%) | 3 (27.3%) |
| Tell-Show-Do | 53 (71.6%) | 2 (22.2%) | 10 (62.5%) | 18 (58.1%) | 2 (66.7%) | 4 (36.4%) |
| Systematic Desensitization | 2 (2.7%) | 0 (0.0%) | 0 (0.0%) | 2 (6.5%) | 0 (0.0%) | 1 (9.1%) |
| Voice Control | 1 (1.4%) | 1 (11.1%) | 2 (12.5%) | 2 (6.5%) | 0 (0.0%) | 0 (0.0%) |
| Modeling | 1 (1.4%) | 0 (0.0%) | 1 (6.2%) | 1 (3.2%) | 1 (33.3%) | 1 (9.1%) |
| CBMT: Conventional behavior management techniques | | | | | | |

**Supplementary Table S2.** Firth's bias-reduced logistic regression models predicting adoption of alternative therapies in dental practice

| **Variable** | **Play area*^1^*** | **Pediatric-themed operatories*^1^*** | **Pyjama color*^1^*** | **Audiovisual tool*^1^*** | **Music*^1^*** | **Aromatherapy*^1^*** | **Breathing relaxation techniques*^1^*** |
| --- | --- | --- | --- | --- | --- | --- | --- |
| Age, years |  |  |  |  |  |  |  |
| ≤ 35 | — | — | — | — | — | — | — |
| > 35 | 0.70 (0.28 - 1.70); 0.434 | 1.24 (0.48 - 3.17); 0.652 | 0.90 (0.27 - 2.77); 0.858 | 1.03 (0.46 - 2.29); 0.948 | 0.62 (0.25 - 1.56); 0.308 | 1.51 (0.45 - 4.59); 0.488 | 2.54 (1.01 - 7.11); 0.049 |
| Sex |  |  |  |  |  |  |  |
| Male | — | — | — | — | — | — | — |
| Female | 1.77 (0.72 - 4.63); 0.215 | 3.36 (1.14 - 12.22); 0.027 | 2.71 (0.56 - 26.78); 0.237 | 2.22 (0.98 - 5.30); 0.057 | 2.43 (1.04 - 5.69); 0.040 | 2.63 (0.58 - 25.02); 0.231 | 1.67 (0.72 - 3.92); 0.236 |
| Dental specialty |  |  |  |  |  |  |  |
| General Dentistry | — | — | — | — | — | — | — |
| Pediatric Dentistry | 5.50 (2.14 - 15.62); <0.001 | 11.38 (4.23 - 34.51); <0.001 | 14.75 (4.38 - 63.58); <0.001 | 2.08 (0.86 - 5.23); 0.104 | 7.42 (2.10 - 39.79); <0.001 | 2.19 (0.69 - 6.98); 0.183 | 6.70 (1.95 - 35.10); 0.002 |
| Orthodontics | 2.20 (0.76 - 6.66); 0.148 | 2.66 (0.85 - 8.23); 0.093 | 7.13 (1.54 - 35.93); 0.013 | 1.12 (0.38 - 3.30); 0.832 | 3.89 (1.06 - 21.21); 0.040 | 2.13 (0.47 - 8.35); 0.307 | 1.04 (0.35 - 3.27); 0.939 |
| Others | 0.35 (0.09 - 1.11); 0.076 | 0.90 (0.24 - 2.86); 0.867 | 1.63 (0.15 - 10.84); 0.642 | 0.85 (0.32 - 2.25); 0.750 | 1.30 (0.48 - 3.71); 0.604 | 0.20 (0.00 - 1.80); 0.179 | 0.77 (0.28 - 2.08); 0.602 |
| *^1^* OR (95% CI); p-value | | | | | | | |
